# Health inequalities in infectious disease modelling

**DOI:** 10.1038/s44528-026-00011-8

**Published:** 2026-06-30

**Authors:** Rosalind M. Eggo, Alexis Robert, Kamlesh Khunti, Hannah E. Clapham

**Affiliations:** 1 Department of Infectious Disease Epidemiology, London School of Hygiene & Tropical Medicine, London, UK; 2 Department of Infectious Disease Epidemiology and Dynamics, Institute of Tropical Medicine, Nagasaki University, Nagasaki, Japan; 3 Diabetes Research Centre, University of Leicester, Leicester, UK; 4 National Institute for Health Research (NIHR) Applied Research Collaboration East Midlands (ARC EM), University of Leicester, Leicester, UK; 5 Saw Swee Hock School of Public Health, National University of Singapore and National University Health System, Singapore, Singapore

**Keywords:** Computational biology and bioinformatics, Diseases, Health care, Mathematics and computing

## Abstract

Infectious disease transmission is unequal, with some groups experiencing long-standing higher burdens of infection and disease. Here, we discuss the urgent need for the field of transmission modelling to develop methods, collaborations and understanding of the drivers and dynamics of infectious disease inequalities to support policymaking that aims to mitigate inequalities.

During the Covid-19 pandemic in England, age, deprivation and ethnicity were quickly identified as major factors associated with higher rates of infection, hospitalisation, and mortality^1,2^. Decision-making and interventions were informed by transmission models, able to simplify complex systems and provide epidemic projections, scenario planning, or long-term burden forecasts. However, although age is routinely included in transmission models, deprivation and ethnicity are not, hence most transmission models cannot be used to understand observed inequalities or to design mitigating interventions.

Though Covid-19 brought these issues into sharp focus, epidemics of many pathogens are shaped by health inequalities in socioeconomic conditions, ethnicity, housing, occupation, access to care and many others. Recognising and incorporating this diversity in transmission models is necessary, and models that ignore these factors risk shaping interventions that ignore and could perpetuate these inequalities.

## Infectious disease transmission creates non-linearities

Transmission is an inherently nonlinear process, where the probability of new infections is modulated by infection prevalence, contact patterns, immunity, interventions, and other factors, many of which are interlinked and vary through time. Transmission models can simplify these complex systems, offering structured ways to anticipate outcomes and evaluate interventions. Because transmission models capture the feedback between groups, they can show how different demographic and social structures shape transmission itself in a way that static models cannot^3^. For instance, transmission models can evaluate whether one group experiences worse outcomes partly because of an age distribution that increases contact rates, which is a different problem - requiring different mitigation strategies - than excess risk driven by housing or occupational exposure. This distinction is critical for policy: it tells decision-makers not just that a community is at higher risk, but why, and therefore what kind of intervention is likely to help.

## Modelling inequities and inequalities is a necessary next step

Well-known differences in social contacts and disease severity have led many existing models to incorporate age and risk groups^4,5^. In most settings, different socioeconomic and ethnic groups exhibit marked differences in key demographics. For instance, in England, individuals who identify as White British are on average older, live in less deprived areas, and in smaller households^6^. This will impact transmission: an older age distribution in one group changes contact patterns; overcrowded housing in another drives higher within-household spreads; low vaccine uptake in a high contact group reduces indirect protection. Severity will also be impacted: chronic conditions cluster along lines of inequality and increase the risk of severe outcomes following infection, which makes some groups not only more likely to get infected but also to get seriously ill if they are infected.

COVID-19 gave the field a vivid illustration of what a lack of inequalities in models looks like in practice. For example, essential workers or people who had to work for economic reasons could not reduce contact in the way that national guidance suggested^7^; people in overcrowded housing faced higher within-household transmission during school closures or lockdowns; communities with longstanding and justified reasons for caution around state institutions engaged differently with vaccination programmes. Each of these is a modellable phenomenon, but most models did not quantify their effects, nor stratify the impact of policies in each group. Incorporating these health inequalities into models does not require a fundamental shift in the field, rather an evolution^8^ towards using modelling tools on this “wicked problem”.

## Understanding the local context is critical for incorporating the intersection of risk factors

Expanding models to explicitly include broader social determinants – such as socioeconomic status, ethnicity, gender, or access to services – is challenging, due to the nature of the variables themselves. Unlike age or biological markers, factors such as ethnicity or deprivation are not straightforward and present key intersectionalities^9^. The distribution of ethnic diversity, socioeconomic status, age, and prevalence of comorbidities often overlaps in ways shaped by historical and cultural factors specific to each context^10^. A group experiencing deprivation that is also predominantly from an ethnic minority background does not experience the simple sum of two risks; the combination generates specific vulnerabilities that neither dimension alone predicts. Even within a country or city, the same epidemic can have profoundly different dynamics depending on housing quality and composition, occupational mix, healthcare access, demography, prevalence of comorbidities and the specific history of trust and the relationship with the state^11^.

Understanding the right model inputs requires deep knowledge of the factors that drive inequality and the lived experience of it. As examples, the reasons for vaccine hesitancy in a specific community, the role of faith institutions in health communication and the practical constraints on isolation for families in overcrowded housing are often known to community health workers, local public health teams, and patient advocates. The field needs to incorporate this knowledge into our models at the outset, and not *post hoc*.

## Data are key to quantifying inequalities and to future modelling

The quality and granularity of stratified data of health outcomes are critical for future modelling, but global heterogeneity in data quality remains one of the central challenges for allowing international comparison in models. Further, the input data also need to be stratified in ways that allow more detailed models to be created and parameterised, for example via social contact data^12^, household size and composition^13^, occupation or other potential drivers.

Broad groupings of ethnic or socioeconomic groups can conceal very different risk profiles: communities sharing a census ethnicity category may have radically different occupational exposures, housing conditions, and relationships with healthcare systems. More disaggregated coding of dimensions of inequalities is useful to models, but there is always a balance with model parsimony. The same applies to socioeconomic data: area-level deprivation measures are valuable proxies, but individual-level data on income, housing type, and employment unlocks a further level of precision.

Crucially, this data infrastructure is not static - linkages between datasets that were difficult or impossible a decade ago are now routine in several countries, and the momentum is towards greater integration, not less. For settings where data infrastructure is still developing, the lesson is clear: investment in comprehensive population-level data collection and linkages, with reliable ethnicity and deprivation coding, is critical for future pandemic preparedness^14,15^. The specifics of the data need to be tailored to each context, and should reflect the inequalities that need action, and the political or legislative will to address them. For example, some countries legally prohibit the collection of ethnicity data^16^, often with stated equity intentions — but without data, inequalities cannot be identified, monitored, or reduced.

## Strengthening connections across disciplines

In England, during the COVID-19 pandemic, a cross-disciplinary advisory group was created with an explicit mandate to monitor and provide evidence on emerging ethnic disparities^17^. The value of this group was not only analytical but aimed to ensure that the right questions were being asked, from data collection through to intervention design and real-time monitoring. Bringing cross-disciplinary perspectives together can significantly enhance modelling efforts, for example, by including insights from social science on which variables are most meaningful, while modelling can help quantify their potential impact. In turn, policymakers benefit from clear, actionable, and timely outputs that are both analytically robust and grounded in lived realities.

Cross-disciplinary collaboration would also support the processing of results and ensure correct communication of conclusions. For example, explicitly including variables like ethnicity in models risks leading to interpretations on biological causation, when in reality such variables act as proxies for structural inequities. Poorly handled models can reinforce stereotypes or obscure underlying drivers such as poverty, discrimination, or unequal access to services. Building connections with community expertise, social scientists, and public health practitioners can produce better models and more trusted responses.

## Designing models with inclusion in mind

A shift is needed so that socioeconomic and ethnic heterogeneities are considered early in model development and in defining the research goal. Compartmental models are fast and tractable but limited in how much individual-level heterogeneity can be incorporated, whereas individual-based models can include detailed personal characteristics but are extremely data-hungry to parameterise. The right choice depends on the research need and understanding the trade-offs.

When epidemic models incorporate the dimensions along which disease burden is unequally distributed, they become capable of answering a qualitatively different set of questions. Not just how large will this epidemic be, but who will bear it, where the pressure points are, and which interventions will reach the people most at risk. The need for these models is clear and does not require a complete rethinking of existing approaches. Rather, the priority should be to build on established practices, enriching them through shared expertise, sustained relationships, and valuing of domain expertise. The modelling community has an opportunity to build methods, collaborations and understanding proactively, before the next crisis, rather than under pressure.

## Data Availability

No datasets were generated or analysed during the current study.
